# Ancestry informative markers and selected single nucleotide polymorphisms in immunoregulatory genes on preterm labor and preterm premature rupture of membranes: a case control study

**DOI:** 10.1186/s12884-016-0823-1

**Published:** 2016-02-05

**Authors:** Bruna Ribeiro de Andrade Ramos, Niele Dias Mendes, Aline Aki Tanikawa, Marcos Antônio Trindade Amador, Ney Pereira Carneiro dos Santos, Sidney Emanuel Batista dos Santos, Erick C. Castelli, Steven S. Witkin, Márcia Guimarães da Silva

**Affiliations:** Department of Pathology, Botucatu Medical School, São Paulo State University – UNESP, Distrito de Rubião Júnior, 18618-970 Botucatu, São Paulo Brazil; Blood Transfusion Center, Botucatu Medical School, São Paulo State University – UNESP, Botucatu, São Paulo Brazil; Department of Genetics, Biological Sciences Institute, Pará Federal University – UFPA, Belém, Pará Brazil; Department of Obstetrics and Gynecology, Weill Cornell Medical College, New York, NY USA

**Keywords:** Preterm labor, Preterm premature rupture of membranes, Inflammatory response, Single nucleotide polymorphisms (SNPs), Ancestry informative markers (AIMs)

## Abstract

**Background:**

A genetic predisposition to Preterm Labor (PTL) and Preterm Premature Rupture of Membranes (PPROM) has been suggested; however the relevance of polymorphisms and ancestry to susceptibility to PTL and PPROM in different populations remains unclear. The aim of this study was to evaluate the contribution of maternal and fetal SNPs in the *IL1B, IL6, IL6R, TNFA, TNFR, IL10, TLR2, TLR4, MMP9, TIMP1* and *TIMP2* genes and the influence of ancestry background in the susceptibility to PTL or PPROM in Brazilian women.

**Methods:**

Case–control study conducted at a tertiary hospital in São Paulo State, Brazil. We included women with PTL or PPROM and their babies (PTL: 136 women and 88 babies; PPROM: 65 women and 44 babies). Control group included 402 mother-babies pairs of term deliveries. Oral swabs were collected for identification of AIMs by fragment analysis and SNPs by Taqman® SNP Genotyping Assays and PCR. Linkage Disequilibrium and Hardy-Weinberg proportions were evaluated using Genepop 3.4. Haplotypes were inferred using the PHASE algorithm. Allele, genotype and haplotype frequencies were compared by Fisher’s exact test or *χ*^2^ and Odds Ratio. Logistic regression was performed. Clinical and sociodemographic data were analyzed by Fisher’s exact test and Mann–Whitney.

**Results:**

PTL was associated with European ancestry and smoking while African ancestry was protective. The fetal alleles *IL10-592*C (rs800872) and *IL10-819*C (rs1800871) were also associated with PTL and the maternal haplotype *TNFA-308*G*-238*A was protective. Maternal presence of *IL10-1082*G (rs1800896) and *TLR2*A (rs4696480) alleles increased the risk for PPROM while *TNFA-238*A (rs361525) was protective. Family history of PTL/PPROM was higher in cases, and time to delivery was influenced by *IL1B-31*T (rs1143627) and *TLR4-299*G (rs4986790).

**Conclusion:**

There is an association between European ancestry and smoking and PTL in our Brazilian population sample. The presence of maternal or fetal alleles that modify the inflammatory response increase the susceptibility to PTL and PPROM. The family history of PTL/PPROM reinforces a role for genetic polymorphisms in susceptibility to these outcomes.

## Background

Spontaneous Preterm Labor (PTL) and Preterm Premature Rupture of Membranes (PPROM) are major contributors to neonatal mortality and serious neonatal morbidity worldwide [1]. Every year more than 10 % of all deliveries around the world are preterm [2–4] and these newborns have a 40-fold increased probability of death and are considerably more prone to major long-term complications such as respiratory morbidities and cognitive delay than their term counterparts [5]. Despite all efforts to identify preventive measures and causative mechanisms, prematurity remains an unresolved issue worldwide.

PTL and PPROM are markedly pro-inflammatory syndromes with complex pathway interactions. Regardless of pathologic or physiologic status of labor, this process is always accompanied by a shift from anti-inflammatory to pro-inflammatory state which mediates the events of myometrial contractility, cervical ripening and rupture of fetal membranes that culminate in birth. In the setting of PTL and PPROM the cascate is prematurely triggered by several disease processes such as intrauterine infection, uterine distension, decidual senescence, maternal stress and cervical diseases [6, 7]. Predictive biomarkers and effective prevention and treatment strategies are yet to be elucidated. It seems clear, however, that a genetic predisposition can contribute to PTL and PPROM. Women with family and/or personal history of such complications are at high risk for their re-occurrence [8–10]. The well documented discrepancy in the rates of these adverse outcomes among different ethnicities and populations [11–13] also reinforces the importance of genetic and environmental variations in the susceptibility to develop PTL and PPROM.

As pro-inflammatory syndromes, PTL and PPROM have been largely associated with infection and the preferential induction of high levels of pro-inflammatory over anti-inflammatory mediators [14–17]. Functional polymorphisms that modify the extent of protein production, activity and/or stability may influence pregnancy outcome [18]. This may be especially relevant if the polymorphic genes code for proteins involved in the triggering of labor in response to infection/inflammation and/or alterations in the extracellular matrix. Many previous studies have already identified associations between single nucleotide polymorphisms (SNPs) located in genes involved in the aforementioned pathways and adverse pregnancy outcomes [19–21].

For instance, a polymorphism located in the promoter region of the gene coding for the inflammatory cytokine interleukin 1β (IL-1β) has already been associated with increased risk for PTL in African-American fetal samples [22]. Likewise, the allele *IL6-174*G has been associated with PTL in women carrying the IL1RN*2 allele [19]. Increased levels of IL-6 are often described in cases of PTL or PPROM [14, 23–25] and the genotype *IL6-174* GG leads to increased production of this cytokine [26, 27]. Similarly, a polymorphism in the intronic region of the gene encoding for the receptor of IL-6 was also associated with PTL by Velez et al. [28]. A polymorphism commonly investigated in women undergoing PTL or PPROM is located at the promoter site of the gene *TNF*, termed *TNF-308* [18, 29–32]. The allele *TNF-308*G leads to increased mRNA transcription and is linked to PTL and PPROM [33]. The gene coding for its receptor is also polymorphic [34]. Anti-inflammatory cytokines such as IL-10 are also important in the context of adverse pregnancy outcomes. Vural et al. observed association between the low producing allele *IL10-1082*A and risk for preeclampsia [35]. In that way, polymorphisms that lead to differential expression of inflammatory or anti-inflammatory genes can be involved in the pathophysiology of prematurity.

As the production of inflammatory mediators is triggered by the activation of the transmembrane Toll-like receptors (TLRs), it is possible that SNPs in the genes that code for TLRs may affect PTL and PPROM pathways. Indeed, Krediet et al. reported increased frequency of homozygosis of polymorphic alleles in *TLR2* in patients with PTL [36] while other authors associated increased risk for this complication with SNPs at *TLR4* [37, 38].

Another critical class of molecules for the development of PTL and especially PPROM are metalloproteinases. These proteases are responsible for the events of cervical ripening and rupture of fetal membranes that happen in both physiologic and premature births [39]. In a study performed by Ferrand et al., infants born to mothers that experienced PPROM presented increased frequency of CA repetition at the promoter region of *MMP9* when compared to term newborns [40]. Polymorphisms located at genes coding for tissue inhibitors of metalloproteinases (TIMPs) have also been implicated in PTL [21].

Most of these findings, however, are controversial [20, 21, 41–43] what demonstrates the importance of standardized methods and reproducible techniques as well as strictly performed evaluation adjusted for potential confounding factors. Additionally, great part of the inconsistencies found in the literature can be due to differences in genetic background and environmental exposures, parameters that vary greatly among distinct populations. Therefore, the repertoire of genes involved in induction of PTL and PPROM remain incompletely elucidated and seem to vary among different populations. Particularly in mixed populations there are few pregnancy outcome-related studies that evaluated the role of SNPs in genes that regulate the inflammatory response and none to specifically analyze the influence of ancestry.

The aim of this study was to evaluate the contribution of maternal and fetal SNPs in the *IL1B, IL6, IL6R, TNFA, TNFR, IL10, TLR2, TLR4, MMP9, TIMP1* and *TIMP2* genes and the influence of the ancestry background in the susceptibility to PTL or PPROM in Brazilian women. These genes and SNPs were selected based on biological plausibility and/or existing evidence in the literature for a role in the pathogenesis of the studied conditions. Here we report association between European ancestry and PTL and increased susceptibility to both PTL and PPROM in the presence of alleles that modify the inflammatory response.

## Methods

### Patients

We conducted an ambispective case–control study of singleton pregnant women who delivered at Botucatu Medical School Hospital (Botucatu – São Paulo, Brazil) between 2003 and 2014. The aforementioned hospital is a tertiary center that provides assistance to 68 cities in the State of São Paulo (Southeast Brazil). The case group consisted of women with PTL with intact membranes or PPROM without other pregnancy complications. We collected buccal swabs from 157 women with PTL and from 114 of their babies, and from 80 women with PPROM and from 63 of their babies. Swab collection from PTL and PPROM patients was performed at their admission, while women were still pregnant. Since a significant number of patients did not live in the city and due to difficulties for collection of babies’ samples during their stay - short stays, newborns admitted into intensive care unit - it was not possible to collect samples from all the babies born to mothers included in the case group. We excluded 14 maternal and 23 babies’ samples that did not have sufficient material for analysis and 22 pairs of maternal and babies’ samples that were found to present systemic diseases (arthritis (1), hipertension (2)), fetal abnormalities (1) or gestational pathologies (preeclampsia (4), gestational diabetes (2), placenta previa (2), intrauterine growth restriction (3), intrauterine infection (2), oligoamnio (2), fetal distress (3)). The final case group consisted of 201 maternal samples (136 PTL and 65 PPROM) and 132 baby samples (88 PTL and 44 PPROM). For the control group we first collected 474 samples from mothers-babies pairs of healthy term deliveries, with no previous history of PTL or PPROM, and then matched the first 402 of these samples that met inclusion criteria and had sufficient material to the case group by newborn gender and maternal age – with a maximum difference of two years. Gestational age was calculated by the last menstrual period and confirmed by first trimester ultrasound. Discrepancies were corrected by the ultrasound result. Sociodemographic data, clinical information and personal and family histories were obtained through a standardized, closed-question questionnaire and by examination of medical records. The study was approved by the Human Research Ethics Committee from Botucatu Medical School (Protocol 3858–2011, FMB, Unesp). All patients enrolled provided written inform consent.

### Clinical Definitions

PTL with intact membranes was diagnosed as the presence of at least two regular uterine contractions every 10 min associated with cervical changes in patients with a gestational age between 20 and 37 incompleted weeks. Tocolysis was successfully achieved in 24.3 % of women from this group (33/136) who delivered after 37 weeks of pregnancy. PPROM was diagnosed by history and physical examination, which included documentation of nitrazine positive pooled vaginal fluid obtained by sterile speculum examination between 20 and 37 incompleted weeks of gestation. In this group only 3 women (4.6 %) had their pregnancies prolonged over 37 completed weeks.

### Genotyping of SNPs

Genomic DNA was extracted from buccal swabs in automated Qiacube equipment using QIAamp® DNA Mini Kit (Qiagen) and DNA quantification was performed by spectrometry using Epoch (Biotek). We evaluated 17 SNPs related with eleven different immune modulatory genes as described in Table 1. The SNPs were genotyped using Taqman® SNP Genotyping Assays and Taqman® Genotyping Master Mix (Applied Biosystems) following manufacturer recommendations. PCR reactions were run in 7500 Real-Time PCR System (Applied Biosystems). As there was no Taqman® assay available to genotype rs3918242 in the *MMP9* gene, the identification of this SNP was performed by PCR-RFLP (Restriction Fragment Length Polymorphism) using the primers MMP9 F 5’- GCC TGG CAC ATA GTA GGC CC-3’ and MMP9 R 5’- CTT CCT AGC CAG CCG GCA TC-3’ for amplification, followed by incubation with the restriction enzyme SphI (Biolabs) [40]. For every SNP evaluated, three clinical samples initially identified by real-time PCR or PCR as wild-type homozygous, mutated homozygous and heterozygous were subjected to direct sequencing and used as positive controls to guarantee the reliability of results. Two negative controls (sterile water) were also included in each run. The reproducibility of results – obtained by repeating 10 % of the samples randomly chosen for all assays – was 99.98 %.

**Table 1:** Identification and localization of genotyped SNPs and set of primers designed for sequencing

Gene	Location	Variable sites (^a^)	SNP location^b^	Primers
*IL1B*	2q14	rs1143627 (−31 T > C)	112836810	IL1b31 F 5’-CCCCTAAGAAGCTTCCACCA- 3’
				IL1b31 R 5’-AAGAGAATCCCAGAGCAGCC- 3’
		rs16944 (−511 C > T)	112837290	IL1b511 F 5’-TGAGGGTGTGGGTCTCTACC- 3’
				IL1b511 R 5’-TGGCTAGGGTAACAGCACCT- 3’
*IL6*	7p21	rs1800795 (−174 G > C)	22727026	IL6 F 5’-TGCACTTTTCCCCCTAGTTG- 3’
				IL6 R 5’-GCCTCAGACATCTCCAGTCC- 3’
*IL6R*	1q21.3	rs2228144	154429203	IL6R1 F 5’-GAGAATGCTGCCCTAATCCA- 3’
				IL6R1 R 5’-GCATTGTCTTCCGGCTCTAC- 3’
		rs2228145 (D358A A > C)	154454494	IL6R2 F 5’-GAGGGGAAGGTTCCTTTGAG- 3’
				IL6R2 R 5’-GAACACCACAGGGCCATC- 3’
*TNFA*	6p21.3	rs361525 (−238 G > A)	31575324	TNF238 F 5’-AATCAGTCAGTGGCCCAGAA- 3’
				TNF238 R 5’-ATCTGGAGGAAGCGGTAGTG- 3’
		rs1800629 (−308 G > A)	31575254	TNF308 F 5’-GAAGCCCCTCCCAGTTCTAG- 3’
				TNF308 R 5’-TCTGGGCCACTGACTGATTT- 3’
*TNFRII*	1p36.3	rs653667 (−24660)	12191751	TNFR 5’-F 5’-GAGTGCAGGCTTGAGTTTCC- 3’
				TNFR 5’-R 5’-GTGTTGTGTGTGCCCCATG- 3’
*IL10*	1q31-32	rs1800872 (−592 C > A)	206773062	IL10-592 F 5’-TGGAAACATGTGCCTGAGAA- 3’
				IL10-592R 5’-GAGGGGGTGGGCTAAATATC- 3’
		rs1800871 (−819 G > A)	206773289	IL10-819 F 5’-TGGTGTACAGTAGGGTGAGG- 3’
				IL10-819 R 5’-GGGAAGTGGGTAAGAGTAGTC- 3’
		rs1800896 (−1082 A > G)	206773552	IL10-1082 F 5’-CAACTGGCTCCCCTTACCTT- 3’
				IL10-1082 R 5’-ATGGAGGCTGGATAGGAGGT- 3’
*TLR2*	4q32	rs4696480	153685974	TLR2 F 5’-CTTGGGTGCTGCTGTAACAA- 3’
				TLR2 R 5’-TGTTATCACCAAGGGAGCAG- 3’
*TLR4*	9q32-33	rs4986790 (Asp299Gly)	117713024	TLR4-299 F 5’-CTCTAGAGGGCCTGTGCAAT- 3’
				TLR4-299 R 5’-TCAATGTGGGAAACTGTCCA- 3’
		rs4986791 (Thr399Ile)	117713324	TLR4-399 F 5’-CAACAAAGGTGGGAATGCTT- 3’
				TLR4-399 R 5’-TCAAATTGGAATGCTGGAAA- 3’
*TIMP1*	Xp11.3	rs2070584	47587120	TIMP1 F 5’-CAACAGCAGCAATGGTCACT- 3’
				TIMP1 R 5’-CTGGCAAGATGTGTGAATGG- 3’
*TIMP2*	17q25	rs2277698	78870935	TIMP2 F 5’-TCCTCCTCCTTGTCTTTCCA- 3’
				TIMP2 R 5’-TAGGAACAGCCCCACTTCTG- 3’
*MMP9*	20q11.2-13.1	rs3918242 (−1562C > T)	46007337	MMP9 F 5’-5’- GCCTGGCACATAGTAGGCCC-3’
				MMP9 R 5’-5’- CTTCCTAGCCAGCCGGCATC-3’

^a^As commonly referred in the literature. ^b^Obtained from dbSNP (NCBI)

### Direct sequencing of controls

We designed pairs of primers for all SNPs to be evaluated (Table 1). After DNA amplification the samples were quantified in agarose gel by comparison with Low DNA Mass Ladder (Invitrogen) and purified using Illustra™ GFX™ Gel Band Purification Kit (GE Healthcare). The purified DNA sample was then amplified using reverse and forward primers separately with BigDye® Terminator v3.1 and, subsequently to the steps of DNA precipitation and resuspension, sequencing was performed in 3500 Hitachi equipment (Applied Biosystems). Sequences were analyzed using BioEdit.

### Identification of AIMs

For the identification of maternal Ancestry Informative Markers, a panel of 61 selected insertion/deletion (indel) variable sites were amplified in conditions as described by Resque et al. [44]. The indel markers that constitute this panel were selected based on the characteristic of exhibiting substantially different frequencies between population from different geographic regions. Then the samples were genotyped using ABI PRISM® 3130 Genetic Analyzer (Applied Biosystems) and the results analyzed using the GeneMapper v3.2 (Applied Biosystems). The ladder ABIGS LIZ-500 (Applied Biosystems) was used as a reference for the identification of each indel. A standard of known size was included in each run to ensure quality control of the analysis. As the admixture model assumes that each individual inherits part of their ancestral markers from ancestral populations, the results were plotted against the three parental populations from our database [45] that constitute the Brazilian population – Amerindian, Western European and Sub-Saharan African – to perform ancestry stratification. For twelve samples from the case group there was not enough material to perform this analysis. The software Structure v2.3.4 with 50,000 burnin length was used to estimate admixture.

### Analysis

Allele, genotype and haplotype frequencies were obtained by direct counting. Linkage Disequilibrium (LD) and expectations under the Hardy-Weinberg proportions were evaluated using Genepop 3.4. Haplotypes were inferred using the PHASE algorithm with final iteraction increased 10 times [46]. Allele and haplotype frequencies were compared by Fisher’s exact test and Odds Ratio and genotype frequencies by *χ*^2^ test and Odds Ratio. Maternal allelic data was adjusted by ancestry and smoking by logistic regression using stepwise backwards. Clinical and sociodemographic data were analyzed by Fisher’s exact test and Mann–Whitney. Additionally, in order to perform a more complete evaluation of the SNPs associated to adverse outcomes, their frequencies in different populations were obtained from the 1000 genomes database [47], the haplotypes were inferred using the software Arlequin 3.5 and the frequencies were compared among populations. The software used were GraphPad® Prism 5.0 and SAS 9.3. A *p*-value <0.05 was considered statistically significant.

## Results

### Sociodemographic data and maternal ancestry

Sociodemographic data are displayed in Table 2. Marital status, self-reported ethnicity, parity and years of education were similar between the groups. Family history of PTL and/or PPROM was less common in the control group when compared to PTL (*p* < 0.001) or PPROM (*p* < 0.001). Smoking was increased among women with PTL (*p* = 0.007) when compared to controls. Newborns of PTL and PPROM mothers had significant lower birth weight and apgar scores then those born at term (Table 3).

**Table 2:** Sociodemographic data

Variables^a^	Control (*n* = 201)	PTL (*n* = 136)	PPROM (*n* = 65)	*p* (Control vs. PTL)	*p* (Control vs. PPROM)
Age (years)	23.9 (±6.1)	22.8 (±6.3)	26.2 (±6.2)		
GA at delivery (days)	278 (273–284)	251 (236–260)	247 (238–254)	*p* < 0.001	*p* < 0.001
GA at PTL/PPROM (days)		241 (224–250)	244 (230–251)		
Marital status					
Single	21.1 % (42/199)	27.6 % (35/127)	19.7 % (12/61)	NS	NS
Married	78.9 % (157/199)	72.4 % (92/127)	80.3 % (49/61)		
Self-reported ethnicity					
White	52.3 % (104/199)	62.7 % (84/134)	60 % (39/65)	NS	NS
Non-white	47.7 % (95/199)	37.3 % (50/134)	40 % (26/65)		
Parity					
Primiparous	48.5 % (97/200)	46.3 % (62/134)	36.9 % (24/65)	NS	NS
Multiparous	51.5 % (103/200)	53.7 % (72/134)	63.1 % (41/65)		
Smoking habits					
Yes	11.9 % (24/201)	23.5 % (31/132)	21.5 % (14/65)	*p* = 0.007	NS
No	88.1 % (177/201)	76.5 % (101/132)	78.5 % (51/65)		
Years of study					
Up until 9 years	23.8 % (45/189)	25.6 % (32/125)	21 % (13/62)	NS	
9 to 12 years	70.4 % (133/189)	72 % (90/125)	75.8 % (47/62)		NS
More than 12 years	5.8 % (11/189)	2.4 % (3/125)	3.2 % (2/62)		
Previous PPROM					
Yes	-	20.8 % (15/72)	24.4 % (10/41)		
No	100 % (102/102)	79.2 % (57/72)	75.6 % (31/41)		
Previous PTL					
Yes	-	43.1 % (31/72)	29.3 % (12/41)		
No	100 % (102/102)	56.9 % (41/72)	70.7 % (29/41)		
Abortion					
Yes	24.8 % (28/113)	34.7 % (25/72)	31.7 % (13/41)	NS	NS
No	75.2 % (85/113)	65.3 % (47/72)	68.3 % (28/41)		
Family history PTL/PPROM					
Yes	23.3 % (24/127)	46.6 % (62/133)	43.1 % (28/65)	*p* < 0.001	*p* < 0.001
No	76.7 % (103/127)	53.4 % (71/133)	56.9 % (37/65)		

*PTL* preterm labor, *PPROM* preterm premature rupture of membranes, *GA* gestational age, *NS* non significant. Variable Age presented as mean (±SD). GA presented as median (25–75 %) and compared by Mann–Whitney. Others variables presented as percentage (total number) and compared by *χ* ^2^. For previous PPROM and PTL only multiparous women were considered and for Abortion only women with multiple gestations. ^a^Comparisons were made between PTL and Controls and between PPROM and Controls

**Table 3:** Fetal data

Variables^a^	Control group (*n* = 201)	PTL group (*n* = 88)	PPROM group (*n* = 44)	*p* (Control vs. PTL)	*p* (Control vs. PPROM)
Weight	3210 (2971–3503)	2552.5 (2210–3000)	2255 (1909–2499)	*p* < 0.001	*p* < 0.001
Gender					
Female	49.3 % (99/201)	51.1 % (45/88)	43.2 % (19/44)		
Male	50.7 % (102/201)	48.8 % (43/88)	56.8 % (25/44)		
Apgar					
1	8 (8–9)	8 (7–9)	8 (7–8)	NS	*p* < 0.001
5	9 (9–10)	9 (8–10)	9 (8–9)	*p* < 0.001	*p* < 0.001
10	9 (9–10)	9 (9–10)	9 (9–10)	*p* = 0.02	NS

*PTL* preterm labor, *PPROM* preterm premature rupture of membranes, *NS* non significant. Weight and Apgar presented as median (25–75 %) and compared by Mann–Whitney. Gender presented in percentage (total number). ^a^Comparisons were made between PTL and Controls and between PPROM and Controls

Median of European, Amerindian and African ancestries from women included in the study are shown in Table 4. European ancestry was increased among PTL when compared to controls (*p* = 0.002) while African ancestry was higher in controls when compared to PTL (*p* = 0.009).

**Table 4:** Ancestry contribution estimates for each group

	European	Amerindian	African
Control (*n* = 201)	0.644 (0.530–0.754)^a^	0.117 (0.082–0.190)	0.178 (0.098–0.339)^a^
PTL (*n* = 127)	0.705 (0.582–0.797)^b^	0.121 (0.074–0.182)	0.141 (0.075–0.279)^b^
PPROM (*n* = 62)	0.677 (0.570–0.800)	0.128 (0.080–0.189)	0.151 (0.079–0.260)

*PTL* preterm labor *PPROM* preterm premature rupture of membranes. Data presented as median (25-75 %) and compared by Mann–Whitney. In the comparison between groups, median followed by different letters (a, b) were statistically different. European - Control x PTL: p = 0.002, African - Control x PTL: p = 0.009

### Genotypes and haplotypes in mothers and babies

Genotype frequencies were under Hardy-Weinberg expectations. We detected associations concerning allele or genotype frequencies for *TLR2*, *IL10* and *TNFA* genes and the studied complications. Regarding *IL1B, IL6, IL6R, MMP9, TNFR, TLR4, TIMP1* and *TIMP2* genes, however, there were no differences between PTL or PPROM and controls.

In maternal samples no alleles or genotypes were associated to PTL when compared to controls. When we compared controls with PPROM, the alleles *TLR2*A (rs4696480) (*p* = 0.007) and *TNFA-238*G (*p* = 0.009) (Table 5) as well as the genotypes *TLR2* AA (*p* = 0.004) and *TNFA-238* GG (*p* = 0.012) (data not shown) were associated with this complication . Regarding the babies’ alleles, *IL10-592*C (*p* = 0.01) and *IL10-819*C (*p* = 0.026) were more frequent in PTL than in controls (Table 6). There was no difference in allele frequencies between PPROM and controls in babies' samples for any SNP evaluated.

**Table 5:** Frequencies for maternal alleles *TLR2 (rs4696480)* and *TNFA-238*

	*TLR2*			*TNFA-238*	
Mothers	A	T	Mothers	A	G
Control (*n* = 201)	0.408ª	0.592	Control (*n* = 201)	0.065ª	0.935
PTL (*n* = 136)	0.423	0.577	PTL (*n* = 136)	0.044	0.956
PPROM (*n* = 63)	0.548^b^	0.452	PPROM (*n* = 64)	0.008^b^	0.992

*PTL* preterm labor, *PPROM* preterm premature rupture of membranes. Data compared by Fisher’s exact test. In the comparison between groups, allelic frequencies followed by different letters (a, b) were statistically different. TLR2: Control x PPROM: p = 0.007; TNFA-238: Control x PPROM: p = 0.009

**Table 6:** Frequencies for babies' alleles *IL10-592 and IL10-819*

	*IL10-592*			*IL10-819*	
Babies	A	C	Babies	C	T
Control (*n* = 199)	0.369^a^	0.631	Control (*n* = 199)	0.624^a^	0.376
PTL (*n* = 92)	0.261^b^	0.739	PTL (*n* = 92)	0.723^b^	0.277
PPROM (*n* = 40)	0.300	0.700	PPROM (*n* = 40)	0.625	0.375

*PTL* preterm labor, *PPROM* preterm premature rupture of membranes. Data compared by Fisher’s exact test. In the comparison between groups, allelic frequencies followed by different letters (a, b) were statistically different. IL10-592: Control x PTL: p = 0.01, IL10-819: Control x PTL: p = 0.026

The test of genotypic disequilibrium indicated the presence of LD among *IL1B, IL6R, IL10, TLR4* and *TNFA* SNPs (p < 0.001 for all). Given that, haplotypes were inferred by probabilistic models as described in methods section. In maternal samples, the haplotype *TNFA-308*G*-238*A was protective against PTL (*p* < 0.001) when compared to controls (Table 7). No association was found between the studied phenotypes and haplotypes in PPROM group or in the babies’ samples. No haplotypes in *IL1B, IL6R, IL10* and *TLR4* genes were associated with PTL or PPROM.

**Table 7:** Haplotype frequencies for the *TNFA* in maternal samples

	*TNFA-308-238*		
Mothers	GG	AG	GA
Control (*n* = 201)	0.838	0.097	0.065^a^
PTL (*n* = 136)	0.812	0.154	0.033^b^
PPROM (*n* = 64)	0.875	0.094	0.031

*PTL* preterm labor, *PPROM* preterm premature rupture of membranes. Data compared by Fisher’s exact test. In the comparison between groups, haplotype frequencies followed by different letters (a, b) were statistically different. Control x PTL: p < 0.001

### Logistic regression models

We used logistic regression in maternal data to postulate different models to analyze the effect of polymorphisms, ancestry and smoking combined. European contribution and smoking increased the risk for PTL while African ancestry was protective against this outcome (Table 8). Regarding PPROM, presence of *IL10-1082G* and *TLR2A* (rs4696480) increased the risk for this complication while the allele *TNFA-238A* was protective (Table 9).

**Table 8:** Logistic regression model comparing the PTL and control groups

Variable	Control (*n* = 201)	PTL (*n* = 136)	OR (CI)	*p*
European ancestry	0.64 (0.53–0.75)	0.70 (0.58–0.80)	13.48 (2.84–64.05)	0.001
African ancestry	0.18 (0.10–0.34)	0.14 (0.08–0.28)	0.10 (0.02–0.53)	0.007
Smoking	11.9 (24/201)	23.3 (31/133)	2.35 (1.25–4.44)	0.008

*PTL* preterm labor, *OR* odds ratio, *CI* confidence interval. Ancestry presented as median (25–75 %), smoking as % (fraction)

**Table 9:** Logistic regression model comparing the PPROM group vs. the control group

Variable	Control (*n* = 201)	PPROM (*n* = 65)	OR (CI)	*p*
*IL10-1082G*	0.346	0.423	2.03 (1.06–3.92)	0.034
*TLR2A* (rs4696480)	0.408	0.548	2.93 (1.42–6.06)	0.004
*TNFA-238A*	0.065	0.008	0.11 (0.02–0.87)	0.036

*PPROM* preterm premature rupture of membranes, *OR* odds ratio, *CI* confidence interval. Data presented as allele frequencies

We also used logistic regression to evaluate whether the time interval between PTL or PPROM initiation and time to delivery (TD) was influenced by the variables SNPs, genetic ancestry and smoking. For this analysis we only considered women with PTL or PPROM and splitted them into two groups: short TD (≤24 h) and long TD (>24 h). Women positive for the allele *IL1B-31T* were at higher risk for short TD while those carrying *TLR4-299G* had a longer TD. Genetic ancestry and smoking did not influence this parameter (Table 10).

**Table 10:** Logistic regression model comparing short latency vs. long latency groups

Variable	Short TD (*n* = 87)	Longer TD (*n* = 79)	OR (CI)	*p*
*IL1B-31* T	0.578	0.447	2.93 (1.40–6.11)	0.004
*TLR4-299* G	0.015	0.070	0.19 (0.05–0.70)	0.013

*TD* time to delivery, *OR* odds ratio, *CI* confidence interval. Short TD: ≤ 24 h. Long TD: > 24 h. Data presented as allele frequencies

Lastly, we thought to compare women who delivered very preterm infants (≤34 weeks of gestation) with those with late preterm neonates (>34 weeks of gestation). For this comparison we only included women that delivered prematurely (births initiated either by PTL or by PPROM) and separated them into very preterm and late preterm subgroups. None of the variables - polymorphisms, ancestry or smoking - could be used to create a model to differentiate very preterm from late preterm subgroups.

## Discussion

### Main findings

European ancestry and smoking increased the odds of PTL while African ancestry was protective. The presence in babies of alleles *IL10-592*C and *IL10-819*C was also associated with PTL. Maternal presence of *IL10-1082*G and *TLR2*A (rs4696480) increased the risk for PPROM while *TNFA-238*A was protective. No fetal alleles were associated with PPROM, possibly because of the small size of this subgroup. Regarding haplotypes, *TNFA-308*G*-238*A was protective against PTL in maternal samples. Family history of PTL and/or PPROM was also associated with these outcomes, and time to delivery was influenced by the presence of *IL1B-31* T and *TLR4-299*G.

### Strengths and limitations

One limitation of our study is that this is a single center study, which, despite the positive effect on homogeneous sampling, may include bias such as social background. The strength is the analysis of ancestry markers in admixed population to stratify ethnicity by unbiased methodology for the first time in the literature.

### Interpretation

Populations are generally mixed, and the Brazilian population is one of the most heterogeneous in the world. As self-reported ethnicity is a poor predictor of genomic ancestry [48, 49], we evaluated AIMs to access the role of ancestry in the studied phenotypes. We observed higher contribution of European-originated markers in the PTL group, which at first seemed unexpected once the literature reports higher rates of this complication in African-descendant women [12, 13]. However, these studies are mainly originated from the USA or Europe, regions with different environments than Brazil. As frequencies of polymorphisms are unevenly distributed among populations, the higher European ancestry among our PTL patients may reflect higher frequencies of SNPs that increase the risk for this outcome in the Southeast Brazilian environment. It has been hypothesized that variations of exposure to microorganisms in distinct ancestral environments may have resulted in a selective pressure that maintained genetic variants that increase survival in response to infectious stimuli [12]. In a new environment, polymorphisms that were advantageous may become detrimental [12, 50]. Thus, one specific allele may induce different responses, i.e. confer resistance or susceptibility, in distinct environmental backgrounds. In this way, more studies to identify such polymorphisms in our population are needed. To date, we are the first to use such unbiased approach to evaluate the influence of ancestry in PTL and PPROM in mixed population.

We also described an increased risk for PTL in women who smoke. Exposure to tobacco during pregnancy is a well-documented risk factor for pregnancy complications [51, 52] and increases the risk for fetal morbidities [53]. Preterm infants are more likely to be born to mothers who smoke [54]. Indeed, the risk of preterm birth attributable to smoking has been estimated as more than 25 % [55]. Chang et al. [56] suggested cessation of smoking during pregnancy as a part of a strategy to reduce preterm birth rate in developed countries.

Regarding PPROM, the presence of *IL10-1082*G and *TLR2*A increased the risk for this outcome. Interleukin 10 (IL-10) is a potent regulator of inflammatory response and altered levels of this mediator are involved in the pathophysiology of PTL and PPROM. Nevertheless, there are contradictory findings regarding the influence of polymorphisms located in its promoter region in the IL-10 expression. Annells et al. associated the low producing haplotype *IL10-1082*A*-819* T*-592*A to the inflammatory events of delivery before 29 weeks of gestation [57] and risk of chorioamnionitis [41] while other authors did not find association between these SNPs and adverse pregnancy outcomes [31, 43]. On the other hand, the high producing *IL10-819*C and *IL10-1082*G alleles have also been implicated in the etiology of complications with an inflammatory signature such as preeclampsia [58], and even delivery before 29 weeks of pregnancy [59], and there are reports that correlate the *IL10-1082*A*-819* T*-592*A haplotype with a reduced risk for small-for-gestational age [60]. In the present we report the association between maternal *IL10-1082*G and PPROM and between presence of *IL10-592*C and *IL10-819*C in babies and PTL. The presence of these alleles may disrupt the balance between pro- and anti-inflammatory cytokines, increasing the risk for PPROM and PTL. It is also worth considering that some of the associations found between SNPs and diseases in genetic studies may be spurious as, as mentioned before, they may reflect differences in the distribution of SNPs in distinct populations and as such are risk markers rather than risk factors. For instance, the allele *IL10-592*C reported here to be more frequent among children born preterm is more common in European populations [47], this type of ancestry was also reported by us to increase the risk for this complication.

Toll-like receptors (TLR) are transmembrane proteins that recognize pathogen-associated molecular patterns and play an essential role in innate immune responses. TLR signaling positively regulates the expression of pro-inflammatory genes. Genetic variants in TLR pathways may alter the susceptibility to early PTL and to neonatal complications such as sepsis and necrotizing enterocolitis in preterm newborns [61, 62]. Our study is the first to report an association between a SNP at *TLR2* and PPROM. Sutherland et al. [63] examined the same SNP in patients with sepsis and observed an association between the A allele and development of sepsis and Gram-positive cultures. Considering the importance of subclinical infection in the setting of PPROM it is possible that the presence of the A allele at position rs4696480 facilitates intraamniotic colonization by gram-positive bacteria activating the inflammatory pathways that culminate in PPROM.

The pro-inflammatory cytokine TNF-α also plays an important role in PTL and PPROM. *TNFA-308*A increases the production of TNF-α and has already been associated with PTL and PPROM [31, 32, 64]. Liang et al. [64] suggested that the presence of at least one *TNFA-308*G allele could be protective against PTL. Consistent with the literature, we report a protective role for the low-TNF-α-producing haplotype *TNFA-308*G*-238*A, located in the promoter region, against PTL and association between the low-producing *TNFA-238A* and decreased risk of PPROM.

Another interesting finding is the association between *IL1B-31*T and short TD and *TLR4-299*G and increased TD. The mutated *IL1B-31*T results in 2-fold increased mRNA production compared to the ancestral allele [65]. Thus, as this variant can lead to increased IL-1β levels, the association between the T allele and short delivery latency is perfectly plausible. Conversely, the *TLR4-299*G allele reduces the responsiveness to LPS in cell cultures [66]. Once the pro-inflammatory cascade triggered by TLR-4 binding is less efficiently induced in these patients they are likely to present longer delivery latency. After the PTL and PPROM pathways are triggered tocolytic treatments currently available are mostly inefficient. In a study of women with an unfavorable cervix who required labor induction, Doulaveris et al. [67] reported that patients with the GG genotype in the *ATG16L1* gene - associated with a decreased capacity to induce autophagy - had a reduced time to delivery. The authors suggest this finding may result from increased production of IL-1β due to impaired autophagy. The identification of specific alleles that contribute to the determination of time to delivery can be of value for clinical practice. Finally, the association between family history of PTL/PPROM and their re-occurrence in the study group reinforces the role of genetic alterations in these outcomes.

## Conclusion

Our findings support a role for functional polymorphisms in immunoregulatory genes in both mothers and babies in the development of PTL and PPROM. Moreover, we encourage the analysis of ancestry markers in pregnancy-related studies to obtain a more accurate panorama in mixed populations. In the future, investigations of specific polymorphisms in combination with ancestry markers may more efficiently predict these adverse outcomes.

## Abbreviations

AIMs: ancestry informative markers
IL: interleukin
LD: linkage disequilibrium
PPROM: preterm premature rupture of membranes
PTL: preterm labor
SNPs: single nucleotide polymorphisms
TD: time to delivery
TIMP: tissue inhibitor of metalloproteinase
TLR: toll-like receptor
TNF: tumor necrosis factor
